# Dietary Intake of Individual (Free and Intrinsic) Sugars and Food Sources in the Spanish Population: Findings from the ANIBES Study

**DOI:** 10.3390/nu9030275

**Published:** 2017-03-14

**Authors:** Emma Ruiz, Paula Rodriguez, Teresa Valero, José M. Ávila, Javier Aranceta-Bartrina, Ángel Gil, Marcela González-Gross, Rosa M. Ortega, Lluis Serra-Majem, Gregorio Varela-Moreiras

**Affiliations:** 1 Spanish Nutrition Foundation (FEN), C/General Álvarez de Castro 20, 28010 Madrid, Spain; eruiz@fen.org.es (E.R.); prodriguez@fen.org.es (P.R.); tvalero@fen.org.es (T.V.); jmavila@fen.org.es (J.M.Á.); 2 Department of Preventive Medicine and Public Health, University of Navarra, C/Irunlarrea 1, 31008 Pamplona, Spain; jaranceta@unav.es or javieraranceta@hotmail.com; 3 Department of Biochemistry and Molecular Biology II, Institute of Nutrition and Food Sciences, University of Granada, Campus de la Salud, Avda. del Conocimiento, Armilla, 18100 Granada, Spain; agil@ugr.es; 4 ImFINE Research Group, Department of Health and Human Performance, Technical University of Madrid, C/Martín Fierro 7, 28040 Madrid, Spain; marcela.gonzalez.gross@upm.es; 5 Department of Nutrition, Faculty of Pharmacy, Complutense University of Madrid, Plaza Ramón y Cajal s/n, 28040 Madrid, Spain; rortega@ucm.es; 6 Research Institute of Biomedical and Health Sciences, Universidad de Las Palmas de Gran Canaria, Facultad de Ciencias de la Salud, C/Doctor Pasteur s/n Trasera del Hospital, Las Palmas de Gran Canaria, 35016 Las Palmas, Spain; lluis.serra@ulpgc.es; 7 Department of Pharmaceutical and Health Sciences, Faculty of Pharmacy, CEU San Pablo University, Urb. Montepríncipe, Crta. Boadilla Km 53, Boadilla del Monte, 28668 Madrid, Spain

**Keywords:** sugar intake, added sugar intake, free sugar intake, intrinsic sugar intake, dietary sources of sugars, ANIBES study

## Abstract

The consumption of total and individual sugars is controversial and little is known about consumption and dietary sources in Spain. The purpose was to examine free and intrinsic sugar intake and food and beverage sources. The ANIBES Study (Anthropometry, Intake and Energy Balance in Spain), a cross-sectional study of a representative sample of the Spanish population (9–75 years old; *n* = 2009) carried out in 2013, was used. Food and beverage records were obtained by a three-day dietary record by using a tablet device. The median total sugar intake was 71.5 g/day (17% Total Energy, TE), the intrinsic sugar intake was 38.3 g/day (9.6% TE), and the free sugar was 28.8 g/day (7.3% TE). Total sugar intake (free and intrinsic) was higher in men than in women for all age groups, although in terms of the contribution to total energy intake, the opposite was observed. Differences were observed for free sugar consumption dependent on age and marked differences (up to two-fold) were observed when considering the percent TE, which was much higher in children and adolescents. For the intrinsic sugar, however, a higher contribution to TE was observed in the elderly. The major sources of intrinsic sugars were fruits (31.8%), milks (19.6%), juices and nectars (11.1%), vegetables (9.89%), yogurt and fermented milk (7.18%), low-alcohol-content beverages (4,94%), bread (2.91%), and sugar soft drinks (2.24%), greater than 90% from diet contribution. As for free sugars, sources were sugar soft drinks (25.5%), sugar (17.8%), bakery and pastry items (15.2%), chocolates (11.4%), yogurt and fermented milk (6.44%), other dairy products (5.99%), jams (3.58%), juices and nectars (2.91%), and breakfast cereals and cereal bars (2.78%), summing up to 90% of the contribution. The present study demonstrates that only a moderate percentage of the Spanish population adhered to the present recommendations for total sugar intake, and urgent efforts are needed to improve diet quality in the youngest populations.

## 1. Introduction

The role of dietary sugars has become an increasing and controversial active public health issue in recent years. The high consumption of added sugars has been ultimately associated with various conditions, such as obesity, risk factors for coronary heart disease (CHD), diabetes, and metabolic syndrome [1]. However, at first, the terminology used to describe sugars has resulted in difficulties with respect to providing comparisons between countries and it also impacts the ability to compare intakes with recommendations, risk factors, or disease endpoints, and with the results of intervention studies. The term ‘total sugars’ includes all mono- and disaccharides, namely glucose, fructose, galactose, lactose, sucrose, and maltose. Added and free sugars differ in the quantity of natural sugars included in their definitions. Therefore, free sugars include sugars naturally present in honey, syrups, fruit juices, nectar juices, and fruit juice concentrates, whereas added sugars only refer to those added during processing. No universally accepted definition for added sugars exists [2]. In addition to various definitions for the term “added sugars”, the World Health Organization (WHO) utilizes the term “free sugars”. Free sugars, as defined by the WHO, refer to monosaccharides and disaccharides added to foods and drinks by the manufacturer, cook, or consumer, and sugars naturally present in honey, syrups, fruit juices, and nectar juices. Intrinsic sugars are defined by the WHO as the sugars incorporated in the structure of intact fruit and vegetables. Free sugars are similar to added sugars, as the term includes all sugars and syrups added to foods; however, free sugars also include sugars naturally present in fruit juices, nectar juices, and fruit juice concentrates. As a consequence, the assessment of added/free sugar intake and compliance with recommendations seems to be extremely difficult. Added sugars are chemically identical to sugar that naturally occurs in food products, and the body cannot distinguish the source of the nutrient and processes the sugar in the same way [3]. Sugar may be added to food products for many reasons, most commonly to add sweetness and enhance the palatability of foods. Another function of sugar within food products is texture enhancement. Finally, sugar may also play a role in food safety by inhibiting the growth of microorganisms at high concentrations [4]. It is also well recognized that excess calorie consumption can lead to weight gain and increased risk of obesity and obesity-related comorbidities. The so-called “empty” calories from solid fats and added sugars play a role in this when consumed in excess and unbalanced in terms of energy expenditure [5]. As a consequence, there is an increasing concern that excessive consumption of added sugars may also contribute to the obesity epidemic worldwide [1,6,7].

In 2010, the European Food Safety Authority (EFSA) published its scientific opinion on dietary reference values for carbohydrates and dietary fiber and was unable to set an upper level for sugar intake as a result of insufficient evidence in relation to body weight, cardiovascular risk factors, type 2 diabetes, and nutrient density of the diet or dental caries [8]. The latter was mainly due to an association with frequency, but not to quantity. The WHO published its updated guideline on free sugars intake for adults and children in relation to body weight and oral health [2]. The recommendations were: (i) a reduced intake of free sugars throughout the life-course (strong recommendation); (ii) the reduction of the intake of free sugars to <10% of the total energy (TE) intake in both adults and children (strong recommendation); and (iii) a further reduction of free sugars to below 5% of the total energy intake (conditional recommendation).

It has also been well stated that to design and implement effective measures to reduce added sugars, their dietary sources must be clearly identified [9,10]. In fact, most food composition tables do not include information on the added and free sugars content of foods, and few countries have reported on individual sugar intakes. Recently, Newens and Walton [11] reviewed current intakes of dietary sugars from national representative dietary surveys across the world. Surprisingly, the so-called added sugars intake in adults was only reported in nine countries and ranged from 7.2% TE in Brazilians aged 10 years and older in 2008–2009 to 16.3% TE in US adults aged 18–34 years in 2007–2008. National dietary surveillance, while having inherent limitations (misreporting, accurate updating of food composition tables at the national level, etc.), provides a way to examine eating patterns and their impact on calorie and nutrient intakes across different populations. The use of newly available methodologies (e.g., real-time recording of eating and drinking events) has been urgently claimed to avoid these difficulties if possible [12,13]. Different dietary surveys have been previously conducted in Spain [14,15,16]. However, no one has approached, to date, energy and nutrient intake using new, more accurate technologies. As a consequence, the ANIBES Study (Anthropometry, Intake and Energy Balance in Spain) was recently completed in a representative sample of all individuals living in Spain (excluding the autonomous cities of Melilla and Ceuta) aged 9 to 75 years, living in municipalities >2000 inhabitants [17,18]. The present study focuses on sugar intake (free and intrinsic) in the Spanish diet for the first time, as well as analyzes food and beverage sources that currently contribute to sugar intake, according to sex and age groups. The latter aim is of particular interest, to provide, firstly, more detailed and accurate information on how the different food and beverage groups and subgroups represent the current market in Spain, but also to drive, in the near future, an adequate labeling of added sugars.

## 2. Materials and Methods

The complete design, protocol, and methodology of the ANIBES study have been described in detail elsewhere [17,18].

### 2.1. Sample

The initial potential sample comprised 2634 individuals, and the final sample comprised 2009 individuals (1013 men, 50.4%; 996 women, 49.6%; 2.23% error and 95.5% confidence interval). In addition, for the youngest groups (9–12, 13–17, and 18–24 years old), a boost was considered in order to have at least *n* = 200 per age group and increase the statistical power of the study (error ± 6.9%). The booster interviews are only analyzed in the context of the analysis of these specific subgroups and not in the context of the analysis of the main random sample. Therefore, the final random sample plus booster was 2285 participants. Sample quotas according to the following variables were: age groups (9–12, 13–17, 18–64, and 65–75 years); sex (men/women); geographical distribution (northeast, Levant, south, central, northwest, Barcelona metropolitan area, Madrid metropolitan area, and Balearic and Canary Islands); and locality size: 2000–30,000 inhabitants (rural), 30,000–200,000 inhabitants (semi-urban), and over 200,000 inhabitants (urban). The final fieldwork was carried out from mid-September to November (three months) 2013, but two pilot studies were previously carried out in order to validate the tools and questionnaires to be employed during the main fieldwork [17,18]. The final protocol was approved by the Ethical Committee for Clinical Research of the Region of Madrid (Spain) (code FEN 2013/31, May 2013). All participants were informed of the protocol and risks and benefits of their participation in the study and a written informed consent was obtained from all the study’s participants.

### 2.2. Food and Beverage Records

Study participants were provided with a tablet device (Samsung Galaxy Tab 27.0, Samsung Electronics; Suwon, Gyeonggi-do, South Korea) to record by taking photos all food and drinks consumed during three days, both at home and outside the home. Photos had to be taken before beginning to eat and drink and again after finishing, so as to record the actual intake. Additionally, a description of the meals (food and drinks consumed and discarded; ingredients expressed in home measures—e.g., a glass of whole milk with a spoonful of sugar), recipes, and brands were also recorded with the device. The ANIBES software was developed to receive information from the field tablets every two seconds, and the database was updated every 30 min. With the use of photographs, descriptions, and all of the collected information, the dieticians/nutritionists codified the foods and beverages and assigned grams following three different cleanings of the data. Foods, beverages, and nutrient intakes were calculated from food consumption records using software (VD-FEN 2.1; Fundación Española de la Nutrición, Madrid; Spain), which is based mainly on Spanish food composition tables [19], with several expansions and updates.

### 2.3. Sugar Intake and Quantification

After energy and nutrients were calculated, an estimation was made of the proportion of “intrinsic sugars” and “free” (“added”) sugars through food product labeling according to their brand, with respect to total sugars obtained from the food composition tables (FCT) [19].

#### 2.3.1. Selection of Food Products and Brands

For each coded food and beverage in the ANIBES Study (a total of 761 of which 327 were fresh with no available label), full labeling of packaged food products recorded was collected in order to be representative of at least >80% of the Spanish market, as a weighted average by sales. Therefore, pictures were taken in retail centers, such as hypermarkets, supermarkets, and convenience stores, at an average of two to seven food products either from traditional manufacturer’s brands and from distribution (supermarket own brand), comprising a total of 1164 food products. For each of those, at least one, and up to four different photographs were obtained in order to obtain precise information about packaging, company, and brand, nutritional labeling and readable ingredient lists (3037 photographs). An additional online survey was also conducted for those products that were not found in the field work (less than 10%).

#### 2.3.2. Classification and Quantification of Sugars in Food

- Foods and beverages with no free sugars (“intrinsic” sugars):
  ○ All of those fresh and unprocessed foods which do not carry labelling and without any added ingredient: most fresh fruits, vegetables, meats, fish, etc.
  ○ Packaged/labelled foods when any kind of free/added sugars not indicated in the list of ingredients.

Understanding of “free sugars” according to Regulation of the European Union 1924/2006 on nutrition and health claims made on foods and 1169/2011 on the provision of food information to consumers, to: monosaccharides, disaccharides, and food used by their sweetening properties, except polyols.

- Foods with added sugars:
  ○ All those packaged/labelled foods for which the ingredients list indicate some form of “free sugars”.

From these above data, the natural/intrinsic sugars content is calculated, based on the content of each of the ingredients in the product, and also through the nutritional composition from the FCT. The above referred amount is subtracted from the total sugars content of the nutritional product labeling. Next, weight percentage for both types of sugars are estimated. The total “free sugars” and “intrinsic sugars” for each food and beverage has been considered the average of the brand or brands collected for the item.

### 2.4. Statistical Analysis

The intake data were grouped into 16 food groups, 38 subgroups, and 761 ingredients for in-depth analysis. Every comparison between groups has been performed by a Student’s *t*-test for independent samples with a 95% confidence interval. In addition, the Kolmogorov–Smirnoff normality test was used to test the normality of the distribution: random sample (2009 participants) and random + booster sample (2285). The random sample is used to show the total sample data and to compare between sexes. To compare age groups and sex in age groups, a booster sample was included in order to expand those age groups less represented in the random sample, as previously explained.

## 3. Results

### 3.1. Sugars Intake and Distribution

The median total daily sugar consumption in the Spanish population was 71.5 g (Table 1), contributing 17.0% of the TE intake (Table 2). The median daily intrinsic sugars consumption was 38.3 g (2.9–179.8 g; min–max) (Table 1) which contributes 9.6% TE, whereas for free sugars it was 28.8 g (0.0–189.8 g; min–max) and 7.3% TE. The total sugar intake (free and intrinsic) was higher in men than in women for all age groups (Table 3), although in terms of the contribution to the total energy intake, the opposite was observed (Table 4). Differences were observed for free sugar consumption and marked differences (up to two-fold) were observed when considering the percent TE as much higher (9.8%–10%) in boys and girls (9 to 17 years) when compared to the elderly (5.1% TE). On the contrary, for the intrinsic sugars a considerably higher contribution to TE was observed with advancing age: 13.0% TE in the population aged 65–75 years and roughly 7.6% TE in the adolescent group (13–17 years) (Table 4).

### 3.2. Dietary Sources of Sugars

The major sources of intrinsic sugars (%) in the ANIBES Spanish population (9 to 75 years) were fruits (31.8%), milks (19.6%), juices and nectars (11.1%), vegetables (9.89%), yogurt and fermented milk (7.18%), low-alcohol-content beverages (4.94%), bread (2.91%), and sugar soft drinks (2.24%), summing up to 90% from the diet contribution (Table 5). Women tend to have a higher proportion of intrinsic sugars from fruits, vegetables, milk and dairy products, usually recognized within the healthy dietary model, and a lower proportion from foods/beverages such as juices and nectar juices, low-alcohol-content beverages, or sugar soft drinks.

The major dietary sources for free sugars (Table 6) in the ANIBES Spanish population were sugar soft drinks (25.5%), sugar (17.8%), bakery and pastry items (15.2%), chocolates (11.4%), yogurt and fermented milk (6.44%), other dairy products (5.99%), jams (3.58%), juices and nectars (2.91%) and breakfast cereals and cereal bars (2.78%), accounting for >90% of the contribution.

**Figure 1:**
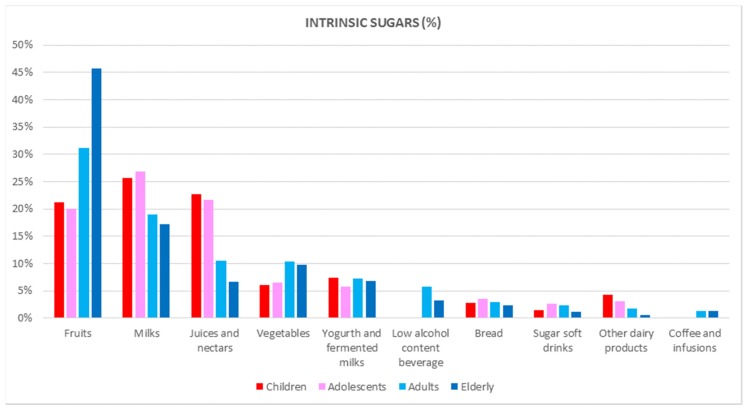
Dietary top 10 food and beverages groups for intrinsic sugars (%) by age group in the ANIBES Study (Anthropometry, Intake and Energy Balance in Spain).

Figure 1 shows the dietary top 10 food and beverage groups contributing to intrinsic sugars (%) according to the different age groups. Marked differences are observed between the youngest (9 to 17 years) and the oldest (65–75 years): much higher for the latter from fruits and vegetables and remarkably lower in the case of milks and juices and nectars. Detailed information of how food and beverage groups and subgroups were contributing to intrinsic sugars consumption in different age groups (9–12; 13–17; 18–64; 65–75 years) and by sex is shown in Table 7, Table 8, Table 9 and Table 10.

**Figure 2:**
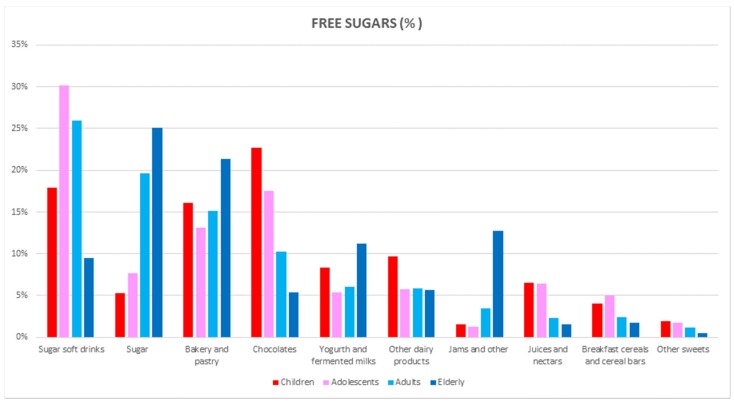
Dietary top 10 food and beverages groups for free sugars (%) by age group in the ANIBES Study.

Figure 2 shows the dietary top 10 food and beverage groups contributing to free sugars (%) according to the different age groups. Sugar soft drinks represented the top source among adolescents and adults, whereas sugar, bakery products and pastries, yogurt/fermented milks and jams are the major contributors in the oldest age group. Other important dietary sources in the 9 to 12 years age group were chocolates (ranked first, 22.7%) followed by sugar soft drinks (17.9%) and bakery products and pastries (16.1%) (Table 11); in adolescents, sugar soft drinks were the main contributor (30.2%), and chocolates (17.6%) and bakery and pastry items (13.1%) ranked next (Table 12). For the older group (adults 18–64 years), sugar soft drinks ranked first (26.0%), followed by sugar (19.7%), bakery and pastry products (15.1%), and chocolates (10.3%) (Table 13). Finally, in the oldest age group (65–75), a different dietary pattern for the free sugars intake from foods was observed (Table 14): sugar (25.1%) was ranked first, closely followed by bakery and pastry items (21.4%), whereas jams and others (12.8%), yogurt and fermented milks (11.2%) and sugar soft drinks (9.46%) ranked next.

## 4. Discussion

In this representative sample of the Spanish population, the total sugar consumption comprised 17% TE (median: 71.5 g/day). The free sugar consumption was 7.3% TE (median: 28.8 g/day) and the intrinsic sugar consumption was 9.6% TE (median: 38.3 g/day). The total sugar intake (free and intrinsic) was higher in men than in women for all age groups, although in terms of the contribution to the total energy intake, the opposite was observed. Differences with age were observed for free sugar and intrinsic sugar consumption when considering the percent of TE: it was much higher for free sugar in children and adolescents compared to intrinsic sugar in the elderly population. One in four Spaniards usually exceed the WHO recommendation that free sugar contribute less than 10% of the total energy intake, according to the present data [2]. Moreover, 25% of the entire population would be within the limits (5% TE) proposed by the SCAN (**Scientific Advisory Committee on Nutrition, United Kingdom**).

In the United Kingdom [20] or the conditional recommendation of the WHO [2]. The groups most likely to exceed the WHO recommendation were, however, children and young people aged 9 to 12 and 13–17 years, which clearly deserves further attention in terms of effort to improve their diet quality. This adherence pattern to the recommendations for the majority of the population from the ANIBES Study is much higher than that found for the Dutch population (29%–33% of the adults 19–69 years) [21] or in the last Australian Health Survey: Consumption of Added Sugars (2011–2012) [22], where one in two Australians (52%) usually exceeded the WHO recommendation. Recently, in a representative national survey in the Netherlands [21], the consumption of total, intrinsic, and added/free sugars was 22% TE, 14% TE, and 12% TE, respectively, much higher than the results obtained in the Spanish ANIBES Study. These results in the Netherlands are also comparable to the recent intake data from the USA and Canada: in 31,305 children and adults aged six years and older from the National Health and Nutrition Examination Survey (NHANES) 2003–2010, added (“free”) sugars provided approximately 14% TE [23,24]; in 35,107 Canadians of all ages from the 2004 Canadian Community Health Survey, the total sugar intake was estimated at 21%TE and added sugar intake at 10%–14% TE [25]. Approximately 13% of adults’ total caloric intakes in the USA came from added sugars between 2005 and 2010 according to National Health and Nutrition Examination Survey (NHANES), which was higher in comparison to the Dietary Guidelines for Americans [24]. Interestingly, Wittekind and Walton [9] have published the trends in sugar intakes reported between 1971 and 2012 in different national nutrition surveys from 10 European countries, but also for Australia, New Zealand, and the United States: In 44 possible comparisons within 13 countries, seven age- and gender-specific or combined groups, and four categories of sugars, the mean population intakes of energy from sugars decreased or remained stable in most comparisons. These findings are also comparable to trends occurring in Spain, according to the Food Consumption Survey evolution in the last decades [16]. In fact, the percentage contribution of the total carbohydrates has steadily decreased since the 1960s in Spain. Moreover, in that decade, the energy profile was in line with the recommendations [16]. In Spain, unfortunately, scarce data or outdated methodology for individual sugars result in a very scarce number of studies to compare with the present results from the ANIBES Study. Of interest, from the ENRICA (Nutrition and Cardiovascular Risk in Spain) study carried out in 2008–2010 (>18 years), it was published that the daily sugar intake per capita was 111.2 g/day, but data on individual sugars were not available [26]. Unfortunately, in the more recent ENIDE (National Dietary Survey in Spain) carried out in 2011 (18–64 years), no data on total and individual sugars were provided [15].

This is the first time that detailed information on the dietary intake of individual sugars and food and beverage sources has been provided in our country. Foods that contributed most to the free sugar intake in the ANIBES Spanish population were sugar soft drinks, sugar, bakery and pastry items, chocolates, yogurt and fermented milk, other dairy products, jams, juices and nectars, and breakfast cereals and cereal bars, accounting for >90% of the contribution. The major sources of intrinsic sugars (%) were fruits, milks, fruit juices and nectar juices, vegetables, yogurt and fermented milks, low-alcohol-content beverages, bread, and sugar soft drinks, summing up to more than 90% of the diet contribution. Nearly 70% of the free sugars were consumed in the total ANIBES Spanish population from usually energy-dense, nutrient-poor foods and beverages, such as sugar soft drinks, sugar, bakery and pastry items, and chocolates. However, marked differences were observed between the age groups for free sugar consumption: in children, the chocolates group ranked first, and sugar was the main contributor in the oldest group (65–75 years), while sugar soft drinks were the first contributor for both adolescents and adults.

Interestingly, summing up the usually perceived healthy foods (fruits, milks, juices/nectars, vegetables, and yogurt and fermented milk) accounted for 80% of the total intrinsic sugars, which was higher in women versus men. For the youngest groups (9 to 17 years), milks ranked first, whereas in adults and the elderly, fruits were the main contributor of intrinsic sugars. Concerning the micronutrient dilution (e.g., vitamins) phenomenon that may occur when the diet model is more based on energy-dense and nutrient-poor foods and beverages, it was found that the sum of the fruits and vegetables contribution (55.4%) to the total intrinsic sugars in the elderly was remarkably higher when compared to the children (27.3%) or adolescents (26.6%).

As published by NHANES [23,24], the top sources of added sugars in Americans are sugar-sweetened beverages, desserts, sugary fruit and candy. Within NHANES, the added sugar intake has already decreased between 1999–2000 and 2007–2008 from 100 g/day (18% TE) to 77 g/day (15% TE), which was primarily due to a reduction in sugar-sweetened beverage consumption. In the already-mentioned national survey in the Netherlands [21], fruit juices and sugar-sweetened beverages, including soft drinks, lemonades, and energy drinks, contributed most to the intake of free sugars, especially in children. In Australian children, sugar soft drinks, cakes, biscuits, pastries, butter-based products, and sugar and sweet spreads were the main contributors to the added sugars intake [22].

Few issues in nutrition generate more scientific controversy than the potential associations between added or free sugars and health. In fact, recently, various scientific and health organizations have recommended upper limits of sugar consumption. The WHO [2], the Scientific Advisory Committee on Nutrition in the United Kingdom (SACN) [20], and the American Heart Association (AHA) [27] have proposed dramatically reducing the upper limits of sugar consumption to levels of 10% of calories consumed or less. The 2015 Dietary Guidelines Advisory Committee [28] also recommended a reduction of the upper limit to no more than 10% of calories from added sugars. This latter recommendation derived also that the Food and Drug Administration (FDA) in the USA recommended a similar upper limit of added sugars consumption (10% TE) and proposed to include such information in the nutrition facts panel [29]. Moreover, a further reduction to below 5% has been suggested by WHO to provide additional health benefits [2]. In contrast, the European Food Safety Administration (EFSA) found no harm, and even some benefit, in fructose consumption comprising up to 25% of total energy [8].

A limitation of the present study is that despite our efforts and innovative methodology, the free sugar content in food products could be under- or over-estimated. Food processes, and consequently the food products available on the market, are continuously changing in content and ingredients. The strengths of the present study include the use of a three-day dietary record using precise and innovative technology to collect food and beverage information at the individual level. As already stated, most food composition tables do not include information on the intrinsic and free sugar content of foods, leading to the use of incomplete food composition tables or supply data. Hence, the development of a food composition table/database for the ANIBES Study including not only the total sugar content, but also intrinsic and free sugars, represents the main strength of the present study. Furthermore, the ANIBES Study was conducted among a representative sample of the Spanish population.

In conclusion, with a mean intake of 17% TE total sugar, 9.6% TE intrinsic sugar, and 7.3% TE added/free sugar, only a moderate percentage of the Spanish population under study adhered to the updated WHO recommendations. However, the results obtained show a remarkably better pattern when compared to other countries. The present findings for the main dietary sources of individual sugars also show a higher variety of foods and beverages contributing, in comparison with non-Mediterranean countries, although urgent efforts are needed to improve diet quality in the youngest populations where patterns and trends are of concern. Future studies are warranted on the associations between the intake of total and individual sugars and health outcomes and chronic diseases in Spain to better clarify nutritional policy.

## Figures and Tables

**Table 1:** Carbohydrates intake and percentiles (P) distribution (g/day) in the Spanish ANIBES Study population (9 to 75 years old).

Nutrients	Mean	Median	SD	SEM	P5	P10	P25	P50	P75	P90	P95	Min.	Max.
**Total Carbohydrates (g)**	185.4	177.4	60.9	1.4	99.4	114.2	143.2	177.4	222.1	267.3	294.9	37.8	450.3
**Starch (g)**	109.1	105.2	40.7	0.9	49.3	61.4	81.7	105.2	132.7	162.4	181.0	15.4	327.8
**Total Sugars (g)**	76.3	71.5	33.9	0.8	30.0	37.3	52.5	71.5	96.2	122.9	136.7	6.7	263.6
**Intrinsic Sugars (g)**	42.4	38.3	22.4	0.5	14.1	18.1	26.2	38.3	53.7	71.5	83.1	2.9	179.8
**Free Sugars (g)**	33.9	28.8	25.2	0.6	3.3	6.6	15.2	28.8	47.3	67.1	83.1	0.0	189.8

**Table 2:** Carbohydrates distribution (% of total energy) in the Spanish ANIBES Study population (9 to 75 years old).

Nutrients	Mean	Median	DS	SEM	P5	P10	P25	P50	P75	P90	P95	Min.	Max.
**Total Carbohydrates (%)**	41.1	41.1	7.2	0.2	29.3	31.9	36.4	41.1	46.1	50.3	52.8	10.8	64.6
**Starch (%)**	24.1	24.1	6.2	0.1	13.9	16.3	20.1	24.1	27.9	31.8	34.3	4.3	49.7
**Total Sugars (%)**	17.0	16.7	5.9	0.1	8.2	9.7	12.8	16.7	20.8	24.6	27.4	2.6	38.6
**Intrinsic Sugars (%)**	9.6	8.7	4.7	0.1	3.6	4.4	6.2	8.7	12.0	15.8	18.5	0.8	29.7
**Free Sugars (%)**	7.3	6.6	4.7	0.1	0.8	1.8	3.8	6.6	9.9	13.7	16.3	0.0	29.1

**Table 3:** Carbohydrates intake and distribution (g/day) by sex and age groups in the Spanish ANIBES Study population.

	Total			Children 9–12 Years			Adolescents 13–17 Years			Adults 18–64 Years			Elderly 65–75 Years		
	Total	Men	Women	Total	Men	Women	Total	Men	Women	Total	Men	Women	Total	Men	Women
***n***	2009	1013	996	213	126	87	211	137	74	1655	798	857	206	99	107
**Carbohydrates (g)**	185.4 (60.9)	200 (64.9)	170.7 (52.7)	214.3 (57.1)	218.2 (61.1)	208.7 (50.7)	224.6 (67.5)	234.5 (70)	206.1 (58.8)	184 (60.4)	198.7 (64.6)	170.3 (52.8)	163.7 (53.4)	175 (59.7)	153.3 (44.7)
**Starch (g)**	109.1 (40.7)	120.4 (42.6)	97.6 (35.1)	122.5 (36.3)	124.3 (37.1)	119.9 (35.3)	134.8 (44.6)	143.2 (45.5)	119.2 (38.5)	109 (40.6)	120.3 (42.8)	98.5 (35.3)	90.6 (32.7)	100.5 (34.5)	81.4 (28.2)
**Total Sugars (g)**	76.3 (33.9)	79.5 (36.6)	73 (30.6)	91.6 (33.3)	93.7 (35.3)	88.4 (30.1)	89.3 (35.1)	90.8 (37.2)	86.6 (31)	74.9 (33.8)	78.4 (36.7)	71.7 (30.5)	73 (34)	74.2 (37.4)	71.8 (30.6)
**Intrinsic Sugars (g)**	42.4 (22.4)	43.2 (23.6)	41.6 (21)	42.9 (19.9)	43.2 (20.5)	42.5 (19.1)	38.5 (20.8)	38.8 (21.5)	37.9 (19.5)	41.6 (22)	43.2 (23.4)	40.1 (20.5)	52.3 (27.4)	52.9 (30.7)	51.7 (24)
**Free Sugars (g)**	33.9 (25.2)	36.3 (27.6)	31.4 (22.3)	48.6 (23.9)	50.5 (25.9)	45.9 (20.6)	50.8 (25.6)	52 (27.8)	48.7 (21)	33.3 (24.8)	35.2 (27.1)	31.6 (22.3)	20.7 (14.9)	21.3 (15.2)	20.1 (14.7)

Results are expressed as the mean ± the standard deviation (in brackets).

**Table 4:** Carbohydrates distribution (% of energy) by sex and age groups in the Spanish ANIBES Study population.

	Total			Children 9–12 Years			Adolescents 13–17 Years			Adults 18–64 Years			Elderly 65–75 Years		
	Total	Men	Women	Total	Men	Women	Total	Men	Women	Total	Men	Women	Total	Men	Women
***n***	2009	1013	996	213	126	87	211	137	74	1655	798	857	206	99	107
**Carbohydrates (%)**	41.1 (7.2)	41 (7.3)	41.2 (7.2)	43.8 (5.8)	43.4 (6.1)	44.4 (5.4)	44.4 (6.9)	43.9 (6.7)	45.2 (7.3)	40.7 (7.2)	40.6 (7.3)	40.9 (7)	40.7 (7.7)	39.6 (7.4)	41.7 (7.9)
**Starch (%)**	24.1 (6.2)	24.7 (6)	23.5 (6.3)	25 (4.7)	24.6 (4.3)	25.4 (5.2)	26.6 (5.3)	27 (5.3)	26 (5.1)	24.1 (6.3)	24.6 (6.1)	23.6 (6.3)	22.5 (6)	22.9 (6)	22.1 (5.9)
**Total Sugars (%)**	17 (5.9)	16.3 (5.8)	17.8 (5.9)	18.8 (5.2)	18.8 (5.4)	18.8 (5)	17.7 (5.6)	16.9 (5.6)	19.2 (5.4)	16.7 (5.8)	16 (5.8)	17.3 (5.7)	18.3 (6.7)	16.7 (6.1)	19.8 (6.9)
**Intrinsic Sugars (%)**	9.6 (4.7)	8.9 (4.3)	10.2 (5)	8.8 (3.7)	8.7 (3.8)	9.0 (3.5)	7.6 (3.8)	7.3 (3.7)	8.2 (3.8)	9.3 (4.5)	8.8 (4.2)	9.7 (4.7)	13 (5.7)	11.7 (5.3)	14.1 (5.7)
**Free Sugars (%)**	7.3 (4.7)	7.2 (4.8)	7.3 (4.6)	9.8 (3.9)	9.9 (4.1)	9.6 (3.8)	10 (4.5)	9.6 (4.6)	10.8 (4.2)	7.1 (4.7)	7.0 (4.8)	7.3 (4.5)	5.1 (3.5)	4.8 (3.1)	5.4 (3.7)

Results are expressed as the mean ± the standard deviation (in brackets).

**Table 5:** Dietary sources of intrinsic sugars (%) from food and beverage subgroups in the ANIBES Study Spanish population aged 9 to 75 years.

Intrinsic Sugars (%)	Total 9–75 Years		
	Total	Men	Women
*n*	2009	1013	996
Fruits	31.8	29.8	33.9
Milks	19.6	18.9	20.2
Juices and nectars	11.1	12.0	10.1
Vegetables	9.89	9.40	10.4
Yogurt and fermented milks	7.18	6.82	7.56
Low-alcohol-content beverages	4.94	6.70	3.09
Bread	2.91	3.29	2.51
Sugar soft drinks	2.24	2.56	1.89
Other dairy products	1.76	2.00	1.50
Coffee and infusions	1.22	1.11	1.34
Ready-to-eat-meals	1.08	1.19	0.97
Pasta	1.06	1.17	0.94
Cheeses	0.82	0.76	0.87
Pulses	0.73	0.76	0.70
Bakery and pastry	0.70	0.68	0.72
Jams and other	0.57	0.42	0.73
Sauces and condiments	0.42	0.46	0.38
Grains and flours	0.33	0.32	0.34
Other drinks (non-alcoholic)	0.32	0.30	0.35
Chocolates	0.30	0.32	0.27
Sausages and other meat products	0.27	0.30	0.23
Breakfast cereals and cereal bars	0.26	0.25	0.27
Appetizers	0.20	0.21	0.20
Other sweets	0.15	0.06	0.25
Non-sweetened soft drinks	0.08	0.06	0.09
Butter, margarine and shortening	0.03	0.03	0.03
Fish and Shellfish	0.03	0.03	0.02
Supplements and meal replacement	0.03	0.02	0.03
Eggs	0.01	0.02	0.01
Olive oil	0.00	0.00	0.00
Water	0.00	0.00	0.00
Sugar	0.00	0.00	0.00
High-alcohol-content beverages	0.00	0.00	0.00
Energy drinks	0.00	0.00	0.00
Sports Drinks	0.00	0.00	0.00
Meat	0.00	0.00	0.00
Other oils	0.00	0.00	0.00
Viscera and spoils	0.00	0.00	0.00

**Table 6:** Dietary sources of free sugars (%) from food and beverage subgroups in the ANIBES Study Spanish population aged 9 to 75 years.

Free Sugars (%)	Total 9–75 Years		
	Total	Men	Women
*n*	2009	1013	996
Sugar soft drinks	25.5	27.9	22.6
Sugar	17.8	16.0	20.0
Bakery and pastry	15.2	14.4	16.1
Chocolates	11.4	11.3	11.5
Yogurt and fermented milks	6.44	6.24	6.68
Other dairy products	5.99	6.59	5.27
Jams and other	3.58	2.72	4.58
Juices and nectars	2.91	3.16	2.62
Breakfast cereals and cereal bars	2.78	2.97	2.56
Other sweets	1.30	0.93	1.74
Sports Drinks	1.14	1.47	0.75
Bread	1.00	0.93	1.07
Ready-to-eat-meals	0.90	1.02	0.77
Sauces and condiments	0.68	0.72	0.63
Energy drinks	0.67	0.95	0.33
Other drinks (non-alcoholic)	0.59	0.48	0.73
Milks	0.59	0.58	0.61
Sausages and other meat products	0.53	0.57	0.49
High-alcohol-content beverages	0.28	0.36	0.18
Fruits	0.23	0.29	0.17
Cheeses	0.19	0.18	0.19
Low-alcohol-content beverages	0.16	0.11	0.22
Grains and flours	0.06	0.04	0.08
Appetizers	0.05	0.06	0.05
Supplements and meal replacement	0.03	0.03	0.03
Meat	0.03	0.03	0.02
Pulses	0.02	0.02	0.03
Vegetables	0.00	0.00	0.00
Pasta	0.00	0.00	0.00
Olive oil	0.00	0.00	0.00
Water	0.00	0.00	0.00
Coffee and infusions	0.00	0.00	0.00
Eggs	0.00	0.00	0.00
Butter, margarine and shortening	0.00	0.00	0.00
Other oils	0.00	0.00	0.00
Fish and Shellfish	0.00	0.00	0.00
Non-sweetened soft drinks	0.00	0.00	0.00
Viscera and spoils	0.00	0.00	0.00

**Table 7:** Dietary sources of intrinsic sugars (%) from food and beverage subgroups in the ANIBES Study Spanish population aged 9 to 12 years: children.

Intrinsic Sugars (%)	Children 9–12 Years		
	Total	Men	Women
*n*	213	126	87
Milks	25.7	27.8	22.6
Juices and nectars	22.7	22.7	22.7
Fruits	21.2	19.3	24.0
Yogurt and fermented milks	7.46	7.34	7.65
Vegetables	6.14	5.84	6.59
Other dairy products	4.22	4.47	3.85
Bread	2.79	2.87	2.68
Sugar soft drinks	1.54	1.68	1.35
Pasta	1.29	1.29	1.29
Ready-to-eat-meals	1.09	1.10	1.08
Bakery and pastry	1.05	0.95	1.20
Cheeses	0.96	1.04	0.85
Chocolates	0.74	0.68	0.83
Pulses	0.60	0.55	0.68
Sauces and condiments	0.60	0.49	0.76
Sausages and other meat products	0.36	0.33	0.41
Grains and flours	0.33	0.31	0.34
Jams and other	0.31	0.24	0.42
Breakfast cereals and cereal bars	0.29	0.29	0.29
Appetizers	0.29	0.40	0.11
Other sweets	0.22	0.12	0.36
Butter, margarine and shortening	0.03	0.03	0.02
Coffee and infusions	0.02	0.03	0.01
Non-sweetened soft drinks	0.02	0.02	0.01
Other drinks (non-alcoholic)	0.02	0.03	0.00
Fish and Shellfish	0.01	0.02	0.01
Eggs	0.01	0.01	0.01
Olive oil	0.00	0.00	0.00
Water	0.00	0.00	0.00
Sugar	0.00	0.00	0.00
High-alcohol-content beverages	0.00	0.00	0.00
Low-alcohol-content beverages	0.00	0.00	0.00
Energy drinks	0.00	0.00	0.00
Sports Drinks	0.00	0.00	0.00
Meat	0.00	0.00	0.00
Other oils	0.00	0.00	0.00
Supplements and meal replacement	0.00	0.00	0.00
Viscera and spoils	0.00	0.00	0.00

**Table 8:** Dietary sources of intrinsic sugars (%) from food and beverage subgroups in the ANIBES Study Spanish population aged 13–17 years: adolescents.

Intrinsic Sugars (%)	Adolescents 13–17 Years		
	Total	Men	Women
*n*	211	137	74
Milks	26.8	29.5	21.7
Juices and nectars	21.7	20.4	24.1
Fruits	20.0	18.6	22.7
Vegetables	6.57	6.51	6.68
Yogurt and fermented milks	5.77	5.43	6.42
Bread	3.50	3.74	3.05
Other dairy products	3.08	3.31	2.64
Sugar soft drinks	2.69	2.89	2.32
Ready-to-eat-meals	1.59	1.61	1.57
Pasta	1.59	1.75	1.30
Bakery and pastry	0.94	0.95	0.93
Cheeses	0.92	0.85	1.05
Pulses	0.74	0.73	0.75
Chocolates	0.70	0.63	0.81
Sauces and condiments	0.58	0.59	0.58
Other sweets	0.54	0.42	0.78
Breakfast cereals and cereal bars	0.44	0.47	0.39
Sausages and other meat products	0.41	0.42	0.39
Appetizers	0.32	0.15	0.64
Grains and flours	0.30	0.31	0.29
Jams and other	0.30	0.28	0.33
Coffee and infusions	0.15	0.19	0.08
Other drinks (non-alcoholic)	0.11	0.05	0.24
Low-alcohol-content beverages	0.10	0.07	0.16
Non-sweetened soft drinks	0.05	0.05	0.04
Butter, margarine and shortening	0.03	0.04	0.01
Eggs	0.02	0.03	0.01
Fish and Shellfish	0.02	0.02	0.02
Supplements and meal replacement	0.00	0.00	0.00
Olive oil	0.00	0.00	0.00
Water	0.00	0.00	0.00
Sugar	0.00	0.00	0.00
High-alcohol-content beverages	0.00	0.00	0.00
Energy drinks	0.00	0.00	0.00
Sports Drinks	0.00	0.00	0.00
Meat	0.00	0.00	0.00
Other oils	0.00	0.00	0.00
Viscera and spoils	0.00	0.00	0.00

**Table 9:** Dietary sources of intrinsic sugars (%) from food and beverage subgroups in the ANIBES Study Spanish population aged 18–64 years: adults.

Intrinsic Sugars (%)	Adults 18–64 Years		
	Total	Men	Women
*n*	1655	798	857
Fruits	31.2	29.4	32.9
Milks	19.0	17.7	20.4
Juices and nectars	10.5	11.6	9.5
Vegetables	10.3	9.77	10.8
Yogurt and fermented milks	7.19	6.97	7.41
Low-alcohol-content beverages	5.84	8.02	3.66
Bread	2.96	3.32	2.60
Sugar soft drinks	2.32	2.74	1.90
Other dairy products	1.72	1.87	1.56
Coffee and infusions	1.35	1.27	1.44
Pasta	1.09	1.20	0.98
Ready-to-eat-meals	1.09	1.16	1.01
Cheeses	0.83	0.78	0.88
Pulses	0.73	0.76	0.70
Bakery and pastry	0.71	0.67	0.76
Jams and other	0.57	0.41	0.73
Sauces and condiments	0.44	0.47	0.41
Other drinks (non-alcoholic)	0.36	0.32	0.40
Grains and flours	0.35	0.34	0.35
Chocolates	0.28	0.28	0.27
Sausages and other meat products	0.28	0.30	0.25
Breakfast cereals and cereal bars	0.27	0.24	0.30
Appetizers	0.20	0.19	0.21
Other sweets	0.17	0.05	0.30
Non-sweetened soft drinks	0.09	0.07	0.11
Supplements and meal replacement	0.03	0.03	0.04
Butter, margarine and shortening	0.03	0.03	0.04
Fish and Shellfish	0.03	0.03	0.02
Eggs	0.01	0.02	0.01
Olive oil	0.00	0.00	0.00
Water	0.00	0.00	0.00
Sugar	0.00	0.00	0.00
High-alcohol-content beverages	0.00	0.00	0.00
Energy drinks	0.00	0.00	0.00
Sports Drinks	0.00	0.00	0.00
Meat	0.00	0.00	0.00
Other oils	0.00	0.00	0.00
Viscera and spoils	0.00	0.00	0.00

**Table 10:** Dietary sources of intrinsic sugars (%) from food and beverage subgroups in the ANIBES Study Spanish population aged 65–75 years: elderly.

Intrinsic Sugars (%)	Elderly 65–75 Years		
	Total	Men	Women
*n*	1655	798	857
Fruits	45.7	45.5	46.0
Milks	17.3	16.8	17.7
Vegetables	9.75	9.74	9.76
Yogurt and fermented milks	6.80	5.63	7.92
Juices and nectars	6.67	7.21	6.15
Low-alcohol-content beverages	3.22	4.76	1.75
Bread	2.40	2.74	2.07
Coffee and infusions	1.27	1.09	1.45
Sugar soft drinks	1.24	0.96	1.51
Jams and other	0.94	0.69	1.17
Pulses	0.67	0.74	0.60
Other dairy products	0.65	0.54	0.77
Ready-to-eat-meals	0.64	0.82	0.48
Pasta	0.53	0.48	0.57
Cheeses	0.53	0.32	0.72
Bakery and pastry	0.41	0.43	0.40
Other drinks (non-alcoholic)	0.41	0.57	0.26
Grains and flours	0.20	0.17	0.22
Breakfast cereals and cereal bars	0.17	0.20	0.14
Sauces and condiments	0.15	0.17	0.13
Sausages and other meat products	0.12	0.17	0.08
Chocolates	0.07	0.11	0.03
Appetizers	0.06	0.07	0.06
Butter, margarine and shortening	0.03	0.03	0.03
Fish and Shellfish	0.02	0.03	0.02
Non-sweetened soft drinks	0.02	0.02	0.03
Other sweets	0.01	0.00	0.02
Eggs	0.01	0.01	0.00
Supplements and meal replacement	0.00	0.00	0.01
Olive oil	0.00	0.00	0.00
Water	0.00	0.00	0.00
Sugar	0.00	0.00	0.00
High-alcohol-content beverages	0.00	0.00	0.00
Energy drinks	0.00	0.00	0.00
Sports Drinks	0.00	0.00	0.00
Meat	0.00	0.00	0.00
Other oils	0.00	0.00	0.00
Viscera and spoils	0.00	0.00	0.00

**Table 11:** Dietary sources of free sugars (%) from food and beverage subgroups in the ANIBES Study Spanish population aged 9 to 12 years: children.

Free Sugars (%)	Children 9–12 Years		
	Total	Men	Women
*n*	213	126	87
Chocolates	22.7	22.1	23.7
Sugar soft drinks	17.9	20.2	14.3
Bakery and pastry	16.1	14.9	18.0
Other dairy products	9.69	9.83	9.47
Yogurt and fermented milks	8.32	8.49	8.05
Juices and nectars	6.57	6.14	7.25
Sugar	5.27	5.08	5.57
Breakfast cereals and cereal bars	4.06	4.07	4.04
Other sweets	1.96	1.56	2.61
Sports Drinks	1.59	2.10	0.77
Jams and other	1.53	1.32	1.86
Ready-to-eat-meals	1.00	1.00	0.99
Bread	0.88	0.79	1.02
Sauces and condiments	0.78	0.67	0.96
Cheeses	0.70	0.83	0.49
Sausages and other meat products	0.62	0.61	0.62
Fruits	0.21	0.19	0.24
Appetizers	0.03	0.04	0.01
Grains and flours	0.03	0.01	0.05
Meat	0.02	0.03	0.02
Pulses	0.01	0.01	0.02
Milks	0.01	0.01	0.00
Other drinks (non-alcoholic)	0.00	0.01	0.00
High-alcohol-content beverages	0.00	0.00	0.00
Vegetables	0.00	0.00	0.00
Olive oil	0.00	0.00	0.00
Water	0.00	0.00	0.00
Low-alcohol-content beverages	0.00	0.00	0.00
Energy drinks	0.00	0.00	0.00
Coffee and infusions	0.00	0.00	0.00
Eggs	0.00	0.00	0.00
Butter, margarine and shortening	0.00	0.00	0.00
Other oils	0.00	0.00	0.00
Pasta	0.00	0.00	0.00
Fish and Shellfish	0.00	0.00	0.00
Non-sweetened soft drinks	0.00	0.00	0.00
Supplements and meal replacement	0.00	0.00	0.00
Viscera and spoils	0.00	0.00	0.00

**Table 12:** Dietary sources of free sugars (%) from food and beverage subgroups in the ANIBES Study Spanish population aged 13–17 years: adolescents.

Free Sugars (%)	Adolescents 13–17 Years		
	Total	Men	Women
*n*	211	137	74
Sugar soft drinks	30.2	31.1	28.3
Chocolates	17.6	16.2	20.2
Bakery and pastry	13.1	13.5	12.4
Sugar	7.66	8.20	6.59
Juices and nectars	6.47	6.05	7.31
Other dairy products	5.74	5.97	5.30
Yogurt and fermented milks	5.36	4.61	6.85
Breakfast cereals and cereal bars	5.04	5.71	3.72
Other sweets	1.72	1.14	2.89
Jams and other	1.24	1.04	1.63
Energy drinks	1.13	1.70	0.00
Ready-to-eat-meals	1.10	1.21	0.89
Sauces and condiments	0.86	0.87	0.85
Bread	0.73	0.71	0.76
Sausages and other meat products	0.45	0.45	0.45
Cheeses	0.41	0.38	0.45
Milks	0.32	0.41	0.14
Sports Drinks	0.32	0.20	0.55
Other drinks (non-alcoholic)	0.21	0.17	0.30
Grains and flours	0.16	0.13	0.22
Fruits	0.12	0.10	0.14
Supplements and meal replacement	0.03	0.05	0.00
Meat	0.03	0.03	0.02
Appetizers	0.01	0.01	0.01
Pulses	0.00	0.00	0.00
Vegetables	0.00	0.00	0.00
Olive oil	0.00	0.00	0.00
Water	0.00	0.00	0.00
High-alcohol-content beverages	0.00	0.00	0.00
Low-alcohol-content beverages	0.00	0.00	0.00
Coffee and infusions	0.00	0.00	0.00
Eggs	0.00	0.00	0.00
Butter, margarine and shortening	0.00	0.00	0.00
Other oils	0.00	0.00	0.00
Pasta	0.00	0.00	0.00
Fish and Shellfish	0.00	0.00	0.00
Non-sweetened soft drinks	0.00	0.00	0.00
Viscera and spoils	0.00	0.00	0.00

**Table 13:** Dietary sources of free sugars (%) from food and beverage subgroups in the ANIBES Study Spanish population aged 18–64 years: adults.

Free Sugars (%)	Adults 18–64 Years		
	Total	Men	Women
*n*	1655	798	857
Sugar soft drinks	26.0	28.3	23.6
Sugar	19.7	18.1	21.3
Bakery and pastry	15.1	14.2	16.0
Chocolates	10.3	9.87	10.7
Yogurt and fermented milks	6.07	6.17	5.97
Other dairy products	5.85	6.47	5.20
Jams and other	3.47	2.63	4.33
Breakfast cereals and cereal bars	2.41	2.36	2.47
Juices and nectars	2.36	2.47	2.25
Sports Drinks	1.42	1.96	0.86
Other sweets	1.20	0.81	1.60
Bread	1.03	0.98	1.08
Ready-to-eat-meals	0.87	0.98	0.75
Other drinks (non-alcoholic)	0.73	0.69	0.78
Sauces and condiments	0.67	0.71	0.63
Milks	0.64	0.61	0.67
Energy drinks	0.62	0.86	0.38
Sausages and other meat products	0.54	0.59	0.48
High-alcohol-content beverages	0.34	0.44	0.24
Fruits	0.24	0.34	0.15
Low-alcohol-content beverages	0.18	0.14	0.22
Cheeses	0.10	0.07	0.13
Grains and flours	0.06	0.05	0.07
Appetizers	0.06	0.07	0.04
Supplements and meal replacement	0.03	0.03	0.03
Meat	0.03	0.03	0.02
Pulses	0.03	0.02	0.03
Vegetables	0.00	0.00	0.00
Pasta	0.00	0.00	0.00
Olive oil	0.00	0.00	0.00
Water	0.00	0.00	0.00
Coffee and infusions	0.00	0.00	0.00
Eggs	0.00	0.00	0.00
Butter, margarine and shortening	0.00	0.00	0.00
Other oils	0.00	0.00	0.00
Fish and Shellfish	0.00	0.00	0.00
Non-sweetened soft drinks	0.00	0.00	0.00
Viscera and spoils	0.00	0.00	0.00

**Table 14:** Dietary sources of free sugars (%) from food and beverage subgroups in the ANIBES Study Spanish population aged 65–75 years: elderly.

Free Sugars (%)	Elderly 65–75 Years		
	Total	Men	Women
*n*	206	99	107
Sugar	25.1	25.2	25.0
Bakery and pastry	21.4	22.8	20.0
Jams and other	12.8	11.1	14.3
Yogurt and fermented milks	11.2	9.41	13.1
Sugar soft drinks	9.46	10.1	8.86
Other dairy products	5.66	4.71	6.60
Chocolates	5.39	7.53	3.28
Breakfast cereals and cereal bars	1.79	1.65	1.93
Juices and nectars	1.51	1.48	1.54
Bread	1.13	0.93	1.32
Ready-to-eat-meals	0.61	0.72	0.49
Other drinks (non-alcoholic)	0.55	0.35	0.74
Milks	0.53	0.80	0.25
High-alcohol-content beverages	0.49	0.98	0.00
Other sweets	0.48	0.50	0.45
Sausages and other meat products	0.47	0.50	0.44
Sports Drinks	0.38	0.52	0.25
Fruits	0.26	0.20	0.33
Low-alcohol-content beverages	0.23	0.04	0.43
Sauces and condiments	0.20	0.19	0.21
Cheeses	0.15	0.15	0.14
Appetizers	0.08	0.02	0.15
Grains and flours	0.07	0.01	0.13
Supplements and meal replacement	0.05	0.09	0.00
Pulses	0.04	0.03	0.05
Meat	0.01	0.01	0.02
Vegetables	0.00	0.00	0.00
Olive oil	0.00	0.00	0.00
Water	0.00	0.00	0.00
Energy drinks	0.00	0.00	0.00
Coffee and infusions	0.00	0.00	0.00
Eggs	0.00	0.00	0.00
Butter, margarine and shortening	0.00	0.00	0.00
Other oils	0.00	0.00	0.00
Pasta	0.00	0.00	0.00
Fish and Shellfish	0.00	0.00	0.00
Non-sweetened soft drinks	0.00	0.00	0.00
Viscera and spoils	0.00	0.00	0.00
